# EEG-Based Person Identification during Escalating Cognitive Load

**DOI:** 10.3390/s22197154

**Published:** 2022-09-21

**Authors:** Ivana Kralikova, Branko Babusiak, Maros Smondrk

**Affiliations:** Department of Electromagnetic and Biomedical Engineering, Faculty of Electrical Engineering and Information Technology, University of Zilina, 010 26 Zilina, Slovakia

**Keywords:** biometry, cognitive load, convolutional neural network, EEG signals, person identification

## Abstract

With the development of human society, there is an increasing importance for reliable person identification and authentication to protect a person’s material and intellectual property. Person identification based on brain signals has captured substantial attention in recent years. These signals are characterized by original patterns for a specific person and are capable of providing security and privacy of an individual in biometric identification. This study presents a biometric identification method based on a novel paradigm with accrual cognitive brain load from relaxing with eyes closed to the end of a serious game, which includes three levels with increasing difficulty. The used database contains EEG data from 21 different subjects. Specific patterns of EEG signals are recognized in the time domain and classified using a 1D Convolutional Neural Network proposed in the MATLAB environment. The ability of person identification based on individual tasks corresponding to a given degree of load and their fusion are examined by 5-fold cross-validation. Final accuracies of more than 99% and 98% were achieved for individual tasks and task fusion, respectively. The reduction of EEG channels is also investigated. The results imply that this approach is suitable to real applications.

## 1. Introduction

Biometric methods have an irreplaceable function in a person’s identification and verification. Due to increases in theft of personal access data to various services, biometry can be considered incomparably secure. Biometry can be defined as a set of methods designed to identify or verify a person according to an individual’s unique physical (physiological) features or habitual (behavioral) traits, and is often used for security reasons. Typical physiological characteristics include fingerprints [1], facial image [2], hand geometry [3], iris [4], or retina [5] patterns. The individual’s behavioral attributes can involve handwritten signature [6], voice [7], gait dynamics [8], and others. Falsification of conventional biometrics data can be overcome by using bioelectric signals such as electrocardiographic (ECG) [9], electromyographic (EMG) [10], electrooculographic (EOG) [11], and electroencephalographic (EEG) [12] signals, for personal identification and verification. These signals contain unique patterns that are difficult to copy or imitate, therefore preserving the secrecy and privacy of the individuals. Consequently, using bioelectric signals as biometric units ensures that the biometric data comes from the competent individual who is genuinely attending the registration of the specific system. This is a fundamental requirement for a biometric mechanism to function properly.

Information obtained by acquiring, processing, and analyzing EEG data is utilized in a wide range of applications. The EEG signals reflect the brain’s electrical activity, and the resultant EEG recording represents the summation of the synchronous activity of many neurons with similar spatial localization. This brain activity can be measured non-invasively by surface electrodes placed on the scalp. The primary EEG signal research focuses on the diagnosis, detection, or prediction of diseases such as epilepsy or epileptic seizures [13,14,15], stroke [16,17], schizophrenia [18], Alzheimer’s disease [19], Parkinson’s disease [20], insomnia [21], etc. Further research is oriented toward the possibility of restoration of the ability of human bodily functions through EEG signals within rehabilitation [22,23,24]. The communication path between the brain and the computer can also be used to control the following objects or devices: virtual cursor [25], keyboard [26], wheelchair [27], or intelligent home [28]. The EEG signals can also be beneficial for quantifying neurological biomarkers of sleep stages [29], advanced driver assistance systems [30], lie detection [31], or in the study of the neurological effect of microwave stimulation [32]. Research interest has recently been concentrated on biometric identification and authentication based on these signals. The main advantage is that EEG signals can only be recorded from a living person, thus it is difficult to steal or falsify them [33,34,35].

The several available experimental configurations for developing a biometric system include different paradigms, feature extraction techniques, or classification algorithms. One paradigm in biometrics involves using a resting state without performing particular mental tasks by the brain when a person remains awake, calm, and relaxed. Other ways are event-related states, including visual or auditory evoked potentials. Other methods involve motor or visual imagery while a person is performing different tasks. Current studies declare a high accuracy rate in recognizing people based on EEG signals.

Ma L. et al. [36] investigated a publicly available database. Data were measured by a 64-channel system during the resting state, namely through closed and open eyes. A convolutional neural network was used for the feature extraction and classification of 10 subjects. The highest classification accuracy belongs to open eyes, 88%, which exceeded the state of closed eyes for all examined scenarios. Fan Y. et al. [37] proposed a personal identification system with a combination data augmentation and convolutional neural network based on resting-state EEG from a public database including 109 subjects. Their system reached an average accuracy of 99.32% using only 14 channels. Sun Y. et al. studied the same public database [38]. In addition to the resting state, four tasks were incorporated both physically and imaginatively, including opening and closing fists and feet. The 1D Convolutional Long Short-Term Memory Neural Network was proposed for EEG-based biometric identification. The results showed a remarkably high recognition rate of 99.58% when using 16 channels.

Moctezuma L. A. et al. [39] created a system for biometric identification of 27 subjects, who performed 33 repetitions of 5 fictional words in Spanish. Extracted features from 14 channels were based separately on a discrete wavelet transform, nine statistical values, and their six combinations (15 in total). A random forest was utilized for feature classification. The maximum classification accuracy of (95 ± 0.4)% was achieved for all channels and subjects based on the instantaneous energy coefficient for each decomposition level from the discrete wavelet transform per channel. Gui Q. et al. [40] presented an identification and authentication framework based on EEG signals recorded while silently reading select words. The EEG signals were collected from 32 subjects via six channels. The noise level was reduced by averaging one channel and a low-pass filter. Wavelet packet decomposition was used to extract delta, theta, alpha, beta, and gamma frequency bands, and then mean value, standard deviation, and entropy were calculated. The feed-forward multilayer neural network was applied for classification. The classification rate, which recognized one subject or a small group of individuals from others, reached approximately 90%. Jayarathne I. et al. [41] proposed an authentication system based on visualizing four-digit numbers while EEG signals were measured. The EEG signals were acquired from 12 subjects using 14 channels. The alpha and beta frequency bands were investigated, whereas common spatial patterns (CSP) values were used as the main features for classification via the linear discriminant analysis (LDA) algorithm. The maximum achieved accuracy reached 96.97%.

Yap H. Y. et al. [42] dealt with biometry based on two EEG acquisition protocols, namely eyes-closed and visual stimulation by words displayed on an LED screen. They created their own database of eight subjects. The eyes-closed protocol achieved the maximum accuracy of 96.42%, while the visual stimulation protocol attained the highest accuracy of 99.06%. Abbas Seha S. N. and Hatzinakos D. [43] investigated the feasibility of using brainwave responses to auditory stimulation for human biometric recognition. The EEG data for the proposed biometric system were recorded from 21 subjects using 7 channels while listening to 4 acoustic tones. Three different features were evaluated based on the EEG sub-band rhythms’ energy and entropy estimation. Extracted features were classified using discriminant analysis with a maximum recognition rate of 97.18%.

Attalah O. [44] proposed a biometric system for person identification based on EEG signals acquired from 36 subjects during multi-tasks. Four features were based on the mean spectral power estimation in delta, theta, alpha, and beta EEG frequency sub-bands for each task. For classification, linear discriminate analysis, k-nearest neighbor, and support vector machine were investigated. The highest accuracy was 100% using a k-NN classifier based on multi-tasks for 14 selected channels. Hema C. R. et al. [45] investigated the brain activity of 50 subjects during relaxation, reading, spelling, and math calculation for person identification. Feed-forward neural network and recurrent neural network were used for classification. The recurrent neural network achieved the highest average accuracy of 95% for the spelling task. Zeynali M. and Seyedarabi H. [46] investigated the performance of an authentication system based on EEG data using a dataset containing EEG data from seven subjects measured by six channels during five mental activities, such as resting state, compose a letter task, math task, geometric figure rotation, and visual counting. Features were extracted using discrete Fourier transform, discrete Wavelet transform, autoregressive modeling, and entropy. The neural network, Bayesian network, and Support Vector Machine were applied to classification. Separately, the influence of only one selected channel on a specific task was examined, where the accuracy rate was in the range of 97.07% to 98.3% using a neural network. The O2 channel was considered optimal without considering the type of mental activity when the accuracy achieved 95%.

This study presents an EEG-based biometric system that includes escalating the brain’s cognitive load, which is the result of relaxing and concentrating on playing a serious game. In this work, we examine the classification of individual tasks (rest state and specific level game) and the fusion of these tasks separately. Specific EEG channels may provide redundant or suboptimal information. Therefore, an essential point for the simplified acquisition, upward the comfort of signal measurement, and reduction of the demands on computing power is to determine and minimize the number of required channels while increasing or maintaining the accuracy of the subsequent classification. For this reason, the influences of different brain regions based on the different electrode selection combinations are investigated. The main contributions of this study can be summarized as follows:

1. This study presents a biometric identification method based on a novel paradigm with unique data for EEG-based person identification. A unique set of EEG data was used, which covers the entire spectrum of the brain load from when the subject was relaxed with closed eyes to solving a difficult task.
2. The ability to identify a person was investigated separately for no brain load, low, medium, and high loads, and the combination of these loads with high accuracy.
3. This research deals with modeling the effects of reducing channel numbers by using a relatively low number of achieved channels, in comparison to person identification accuracy with other studies using a larger number of channels. This can lead to a reduction in cost and time funding in the real-life adoption of the proposed approach.

## 2. Materials and Methods

The EEG data was measured by the BIOPAC MP36 acquisition system (BIOPAC Systems Inc., Goleta, CA, USA), including the precise built-in universal amplifiers and 24-bit A/D converters for signal acquisition. One MP36 unit is capable of sensing four channels. This study used two synchronized MP36 units to measure eight channels. A regular EEG cap with 19 electrodes pre-positioned in the International 10–20 montage was used. The cap is made from Lycra-type fabric with recessed tin electrodes. A more detailed description of the measurement system is provided in [47].

**Figure 1:**
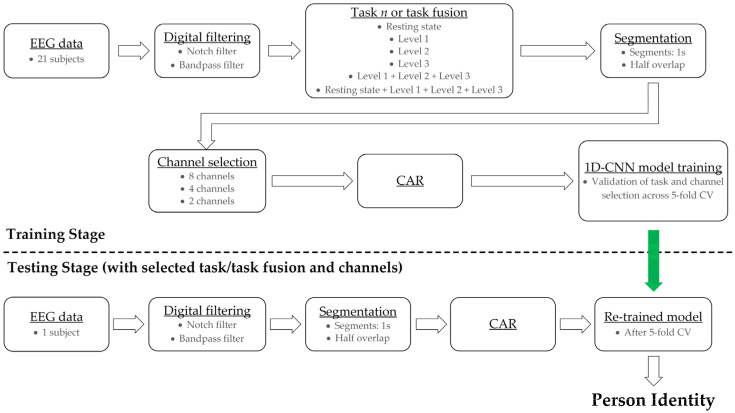
Block diagram of presented attitude in order to determine person identity. The approach is established by the training and testing stage of the 1D-CNN model inclusive EEG data pre-processing. The green arrow depicts the transition from the training to the testing stage while data handling.

The entire presented approach can be summarized into two stages, namely the training and testing stages of the 1D-CNN model, as shown in Figure 1. The training stage includes digital filtering of the raw EEG data (notch and band-pass filter), task selection (resting state, three individual levels, level fusion or game playing, and fusion of all tasks), data segmentation (into segments with a length of 1 s or 1000 samples and half overlap), channel selection (according to recorded scalp regions into eight, four, or two channels), common average re-reference based on available channels, and 1D-CNN model training, validation, and evaluation based on 5-fold CV. The testing stage focuses on an already selected task or task fusion and selected channels. Digital raw data filtering, segmentation, and common average re-reference are preserved. This phase is responsible for determining a person’s identity. Each block is described in the following subsections.

### 2.1. EEG Data

**Figure 2:**
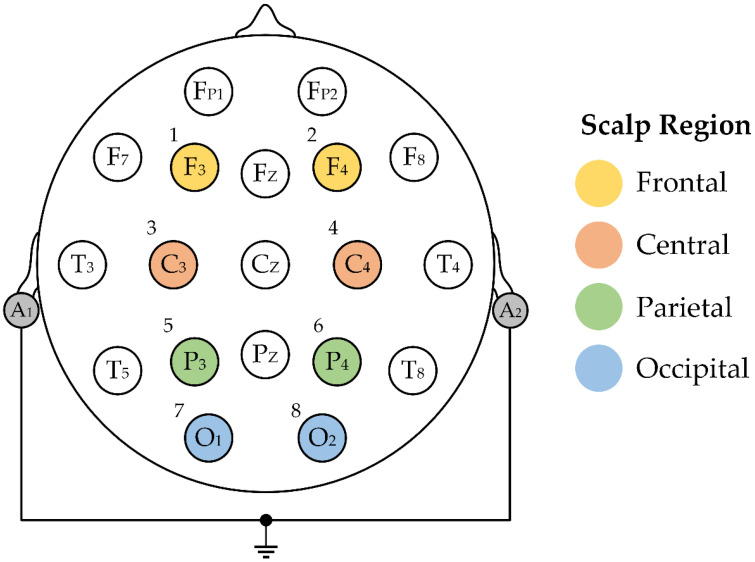
Unipolar electrode configuration for EEG signal measurement, modified according to [47].

This study investigates a database consisting of EEG signals from adult subjects previously analyzed in [47]. The EEG database was primarily created to investigate brain activity using spectral analysis while playing a serious game with rising difficulty. In total, EEG measurements of 21 university students (9 males and 12 females) with a mean age of 22.7 years (range: 21–26 years) are included in the database. The EEG signals were measured by eight unipolar channels with a sampling rate of 1 kHz. Individual electrodes were placed on the scalp, as illustrated in Figure 2. The electrode locations include frontal (F3, F4), central (C3, C4), parietal (P3, P4), and occipital (O1, O2) regions in accordance with the international 10–20 scheme. The reference electrode was constituted by connected and grounded auricle electrodes.

The EEG signals were acquired for each subject during the resting state and while playing a serious game. Individual tasks can be divided as follows:

1. Task 1: Close eyes for approximately 60 s;
2. Task 2: The first level (easy) of the serious game;
3. Task 3: The second level (medium) of the serious game;
4. Task 4: The third level (hard) of the serious game. Eight students were unable to complete this level, so the end of the game was subsequently considered the end of this task in all cases.

### 2.2. Serious Game

A serious game can be defined as the innovative and exciting use of games or gaming elements for purposes more serious than mere “entertainment.” They are beneficial in training cognitive abilities, short- or long-term memory, physical training, and both prevention and physical rehabilitation [47]. The serious game design was focused on the students’ cognitive training in logical thinking. It was realized through logical puzzles with principles based on Boolean algebra. The game is divided into three levels. Each represents a particular difficulty level—easy, medium, and hard—which reflects the escalating cognitive load for the game player.

### 2.3. Data Pre-Processing

The first filtering stage was composed of a digital notch filter to reduce the 50 Hz powerline interferences and a band-pass filter with cut-off frequencies of 0.5 Hz and 95 Hz to eliminate DC offset and unwanted high-frequency noise (including power line harmonics). The majority of the defined frequency band of the EEG signal was covered [48], including the potentially present gamma band (from 30 Hz to 100 Hz) characteristic for the cognitive load [49]. The band-pass filter was the result of Butterworth low-pass and Butterworth high-pass filter cascading.

Subsequently, the common average reference (CAR) method was applied to filtered data to remove the mutual information from all simultaneously recorded channels to increase the signal-to-noise ratio (SNR). It can be calculated as [50]:

(1) $$V_{i}^{\mathrm{CAR}}=V_{i}^{\mathrm{ER}}-\frac{1}{N}\sum_{j=1}^{N}V_{j}^{\mathrm{ER}},$$

where $V_{i}^{\mathrm{CAR}}$ represents modified voltage values of *i*-th EEG signal (channel) after CAR, $V_{i}^{\mathrm{ER}}$ represents the voltage between the *i*-th detecting electrode and the reference electrode, and *N* is the number of all detecting electrodes.

### 2.4. Dataset Preparation

The EEG signals based on the duration of a particular task were segmented in the time domain using a sliding window into segments (epochs) with a fixed length of 1000 samples (1 s) and a half-overlap of 500 samples (0.5 s) to create a set of data for the subsequent classification process.

**Figure 3:**
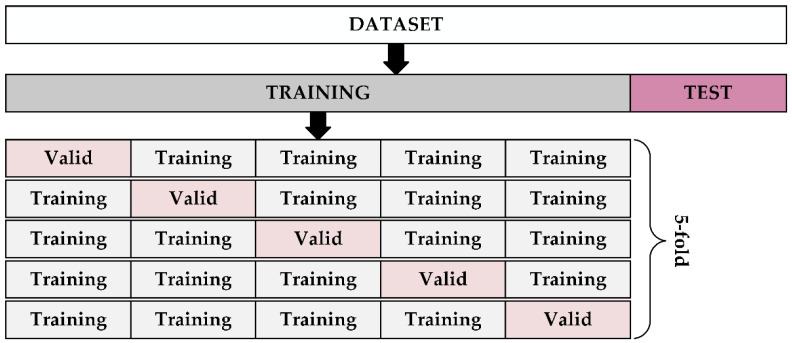
Dataset division according to the 5-fold cross validation.

A 5-fold cross-validation (CV) was used to estimate the predictive performance of the classification model on unknown data with different segment lengths. First, the entire dataset was split at a ratio of 0.8 for training data (for 5-fold CV) and 0.2 for test data (for overall testing of the most accurate case). Next, the training data was divided into five subsets of the same size. Four subsets of data were considered as training sets and the remaining subset as the validation set. A total of five iterations of training were performed, with each iteration corresponding to a unique training and validation data set. The variant of the proposed model with the best accuracy for the corresponding segment length was finally trained and evaluated on test data. The division of the dataset is depicted in Figure 3. In order to reduce the bias of the proposed model’s variants, the 5-fold cross-validation has been incorporated within the training stage. The subjective bias in data division has been minimalized by precise EEG data labeling (resting stage, start, and end of individual game levels) in real-time by the technician during the data acquisition. In addition, the EEG data acquisition was performed while a synchronized camera recorded, which has been used for offline data labeling. Moreover, no subjects involved in this study have been excluded in order to reduce the subjective bias of the proposed model, even when they could not successfully finish the hardest game level. A more detailed description of the measurement setup and protocol is provided in [47].

The z-score method was used for channel-wise data normalization across all training samples, where the mean value from the individual data point *x* was subtracted and divided by standard deviation. The normalized value *x*′ can be defined as:

(2) $$x^{\prime }=\frac{x-\mu }{\sigma },$$

where *µ* denotes the mean value and *σ* denotes standard deviation.

### 2.5. Feature Extraction and Classification

Traditional approaches in EEG-based biometric systems include manually extracted features and conventional classification algorithms such as linear discriminant analysis (LDA) [51,52], k-nearest neighbors (k-NN) [44,53], or support vector machine (SVM) [54,55]. Alternatively, deep learning (DL) algorithms [37,38,56,57,58,59,60,61,62,63] are becoming state-of-the-art in person identification and authentication. These algorithms are able to automate the feature extraction process and eliminate some of the manual human encroachment.

**Figure 4:**
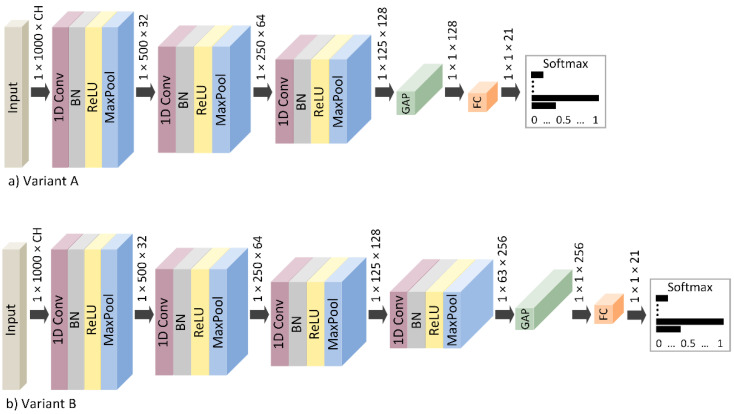
The proposed 1D-CNN model: (**a**) Variant A with three convolutional blocks, (**b**) Variant B with four convolutional blocks.

In this work, a one-dimensional convolutional neural network (1D-CNN) is proposed to automatically extract and classify the most unique neurological features that correspond to the individual subjects. Specifically, two variants of the 1D-CNN model were created using Deep Learning Toolbox in MATLAB R2021b (Mathworks, Inc., Natick, MA, USA). The first variant (hereafter referred to as variant A) contains three convolutional blocks, and the second variant (hereafter referred to as variant B) contains an additive fourth convolutional block. The overall network architectures are depicted in Figure 4.

The input layer receives multichannel one-dimensional EEG signals with a segment length of 1000 samples, which are processed independently via multichannel kernel filters in the first convolutional layer. Variant A obtains 3 convolutional layers with 32, 64, and 128 filters. Variant B includes 4 convolutional layers with 32, 64, 128, and 256 filters. Each convolutional kernel performs convolutional operations on local regions with a kernel size of 1 × 5 and extracts certain features from the input data. Afterward, the Batch Normalization (BN) layer was implemented between the convolutional and activation layers. BN layer can speed up convergence and improve the performance and stability of the neural network. The nonlinear function Rectified Linear Unit (ReLU) was applied as an activation function in convolutional blocks, which changed negative values to 0 and left positive unchanged. The Max Pooling layers were included as the last layer within each convolutional block. These layers were utilized as a downsampling operation to calculate the maximum value over the local pooling regions with a size of 1 × 2 and stride 1 × 2, which reduced the feature maps’ dimension. Instead, a typical fully connected layer, the Global Average Pooling (GAP) is included after the convolutional blocks and produces the temporal average of the feature map from the previous layer. The output layer was a fully connected layer containing 21 neurons corresponding to the number of output classes. The activation function softmax was applied to this layer. The softmax function assigns the probability to each class with which the given input belongs to the respective class. The class with the highest probability is considered the correct result of the classification.

The Adam optimization algorithm and cross-entropy loss function are used to train the proposed 1D-CNN model. The model was trained for a maximum of 100 epochs with a mini-batch size of 64 and a constant learning rate of 0.001. The training data were randomly shuffled before every epoch. The output network was considered the network that achieved the best loss validation during 5-fold cross-validation.

### 2.6. Evaluation Metrics

The classification model performance was evaluated on selected statistical measures: *Average Accuracy*, *Macro Average Precision*, *Macro Average Recall*, and *Macro Average F1 score* for the multiclass problem. They can be defined as follows [64]:

(3) $$Average\;Accuracy=\left(\overset{K}{\underset{k=1}{\sum }}\frac{TP_{k}+TN_{k}}{TP_{k}+TN_{k}+FP_{k}+FN_{k}}\right)\times \frac{100\%}{K},$$

(4) $$Macro\;Average\;Precision=\left(\overset{K}{\underset{k=1}{\sum }}\frac{TP_{k}}{TP_{k}+FP_{k}}\right)\times \frac{100\%}{K},$$

(5) $$Macro\;Average\;Recall=\left(\overset{K}{\underset{k=1}{\sum }}\frac{TP_{k}}{TP_{k}+FN_{k}}\right)\times \frac{100\%}{K},$$

(6) $$Macro\;Average\;F1\;score=2\times \frac{Macro\;Average\;Recall\times Macro\;Average\;\mathrm{Precision}}{Macro\;Average\;Recall+Macro\;Average\;\mathrm{Precision}},$$

where *TP* is a true positive, *FN* is a false negative, *FP* is false positive, *k* is a particular class, and *K* is a number of all classes. *Average Accuracy* is the percentage of correct prediction for total observations and was considered a fundamental metric. *Macro Average Precision* is the average percentage of correctly predicted positive observations to the total predicted positive observations. *Macro Average Recall* is the average percentage of correctly predicted positive observations to all observations. *Macro Average F1 score* is the weighted average of *Macro Average Precision* and *Macro Average Recall*.

## 3. Results and Discussion

This section compares the performance of both variants of the proposed 1D-CNN model. Table 1, Table 2, Table 3, Table 4 and Table 5 show the results for the individual states, corresponding to the increasing brain load and fusion of these states on the validation subset of data. Variant performances were evaluated based on the achieved *Average Accuracy*, *Macro Average Precision*, *Macro Average Recall*, and *Macro Average F1 score*. The impact of channel reduction and classification model on person recognition is investigated using a 5-fold CV. For this reason, all the subsequent results are given in the form of mean value ± standard deviation. 

The results are shown for all eight channels and the reduced number of channels. Systematic channel reduction is performed based on an individual measured region’s combination of the scalp. Each region is constituted by 2 channels (see Figure 2). In the first step, the number of channels is halved, i.e., in two regions, where 6 combinations are created: frontal (F) + central (C), frontal (F) + occipital (O), frontal (F) + parietal (P), central (C) + occipital (O), central (C) + parietal (P), and parietal (P) + occipital (O) region. In the second step, the number of channels is limited to a quarter of the original number, where each region is examined individually, i.e., through four combinations. A total of 104 different cases are investigated.

Table 1 presents the results for person identification based on the resting state with closed eyes. For all channels, an average accuracy rate of (99.17 ± 0.41)% and (99.46 ± 0.44)% are achieved for variant A and variant B, respectively. In half of the channels, the combination of the central and occipital region shows the highest accuracy of (95.18 ± 1.25)% for variant A and (97.52 ± 1.01)% for variant B. However, a significant decline can be observed when using only one region, where, for the most successful central region, a decrease of up to (34.99 ± 1.64)% is found for variant A and (27.69 ± 0.04)% for variant B compared to the combination of the central + occipital region.

The results of person identification during the first level of the serious game are reported in Table 2. The average accuracy of person identification is (98.99 ± 0.45)% for variant A and (99.61 ± 0.24)% for variant B using eight channels. The highest average accuracy, (94.21 ± 1.32)% for variant A, belongs to the central + occipital region, and (96.71 ± 0.67)% for variant B corresponds to the central + parietal region. For the single region, the average accuracy is less than 65% in all cases.

Table 3 summarizes the results of person identification during the second level of the serious game. As can be seen, the average accuracy of person identification reaches (99.41 ± 0.41)% for variant A and (99.72 ± 0.13)% for variant B when all channels are used. As in the majority of previous results, reducing channels to two regions is most successful for the central + occipital region. The average classification accuracies are (95.62 ± 0.78)% and (97.50 ± 0.73)% for variant A and variant B, respectively. In the case of a single region, the best results can be observed in the occipital region, but with a significant decrease of (61.42 ± 2.05)% for variant A and (67.80 ± 1.71)% for variant B compared to the more examined regions.

The results of person identification according to the third level of the serious game are reflected in Table 4. Including all channels, average accuracy rates of (98.33 ± 0.18)% for variant A and (99.20 ± 0.16)% for variant B are achieved. In half of the channels, the highest average accuracy reached (93.14 ± 0.83)% in the central + parietal region for variant A, and the highest accuracy rate of (95.84 ± 0.45)% corresponds to the frontal + parietal region for variant B. For the single region, the average accuracy does not exceed 65% in all cases.

Furthermore, person identification is investigated based on the fusion of EEG signals recorded while playing the serious game with increasing difficulty (levels 1–3). Based on the present results for the individual levels, three combinations of two scalp regions are selected: frontal + parietal, central + parietal, and central + occipital. Two-channel regions are further excluded. The values of the individual classification metrics are given in Table 5 for this scenario. For all channels, the average accuracy is (98.82 ± 0.29)% and (97.84 ± 0.18)% for variants A and B, respectively. For the two regions, the highest precision rate is achieved by the central + parietal regions with a value of (89.88 ± 1.06)% for variant A and frontal + parietal with a value of (92.58 ± 0.49)% for variant B.

The last evaluated scenario represents a fusion of resting-state and all game levels. The same combination of scalp regions as in the previous case is considered. The average accuracy corresponding to variant A is (98.08 ± 0.30)% and (98.97 ± 0.12)% to variant B for all channels, see Table 6. The highest accuracy for the reduced channels can be observed for the central + parietal combination, namely (91.01 ± 0.77)% and (93.39 ± 0.42)% for variant A and variant B, respectively.

**Figure 5:**
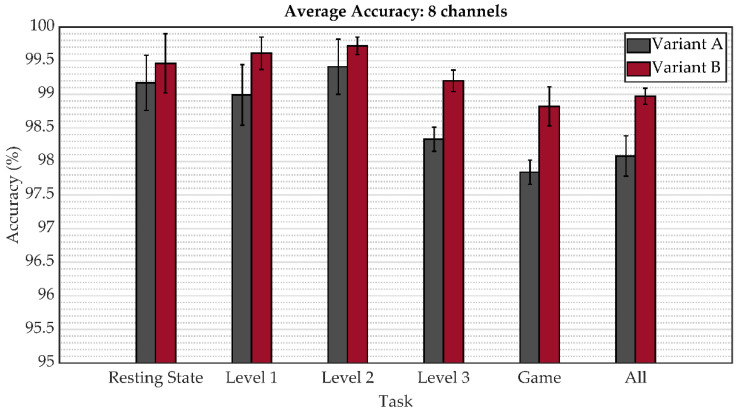
The average accuracy comparison across the model variants for tasks and task fusion corresponding to the eight considered channels.

**Figure 6:**
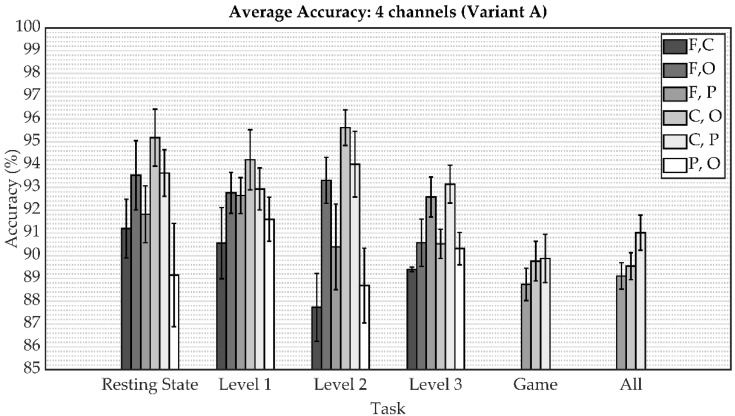
The average accuracy comparison for variant A involving tasks and task fusion corresponding to the reduced channels.

**Figure 7:**
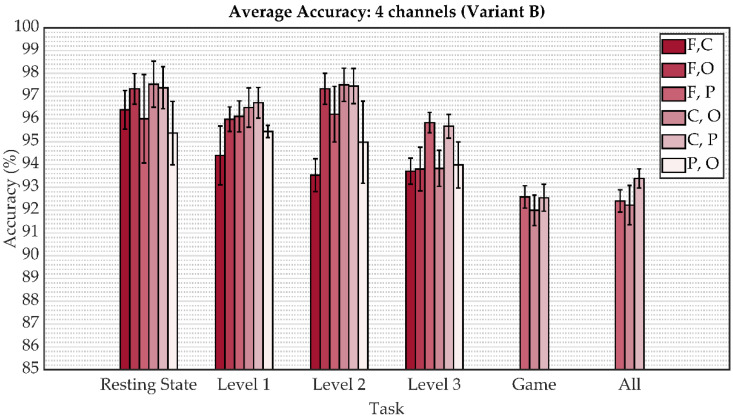
The average accuracy comparison for variant B involving tasks and task fusion corresponding to the reduced channels.

The following figures depict a graphical representation of the aforementioned results in the form of a bar graphs with the indicated standard deviation. The comparison of the average accuracy of both model variants for person identification when all eight channels were applied is shown in Figure 5. These results are shown separately for the resting state, playing a particular game level, playing all game levels, and all states together. The subsequent two figures show this metric for reduced regions from four to two (from eight channels to four channels), specifically in Figure 6 for variant A and Figure 7 for variant B. As can be seen, the results indicate an increase in accuracy on the validation subset of person identification data among 21 people by an additive fourth convolutional block, thus increasing the complexity of the model.

**Figure 8:**
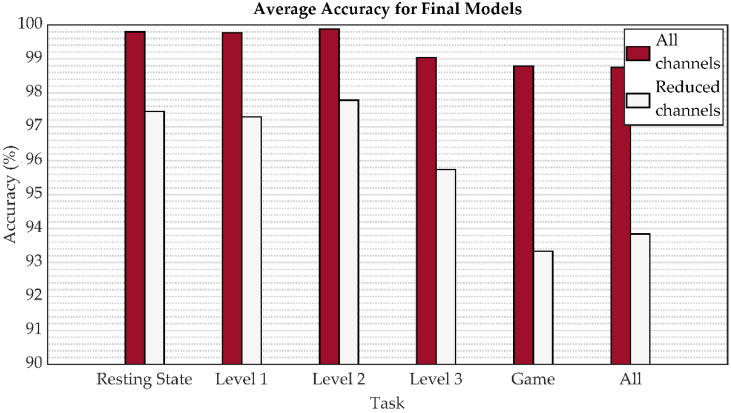
The accuracy evaluation for the final model based on the test data for individual tasks and task fusions.

After evaluating the prediction performance of two variants of the 1D-CNN model with different combinations of input EEG channels, all data from the 5-fold CV are used to train the final model corresponding to each mental task. Based on the previous results, only hyperparameters of variant B are subsequently considered. The final model is rated on an original test data subset. The corresponding classification metrics for the final model evaluation of individual tasks and task fusions are presented in Table 7. Using all channels, individual tasks achieve an average accuracy of more than 99% and task fusions of more than 98% when identifying 21 persons. When the channels are halved, more than 97% average accuracy is obtained for resting state, level 1, and level 2. In the case of level 3, the accuracy is 95.74%. The decrease in the accuracy of this level compared to level 1 and level 2 can be attributed to its complexity and inability to complete for all subjects. This fact was influenced by the diverse brain activity of the individual during the duration of level 3, when there was a gradual lack of concentration caused by human frustration at the individual’s inability to complete level 3. A decrease in accuracy can be seen in the reduction of channels for task fusion, specifically 93.33% and 93.84% for the playing game task and all task fusion, respectively. A graphical representation of the achieved accuracy results is presented in Figure 8.

**Figure 9:**
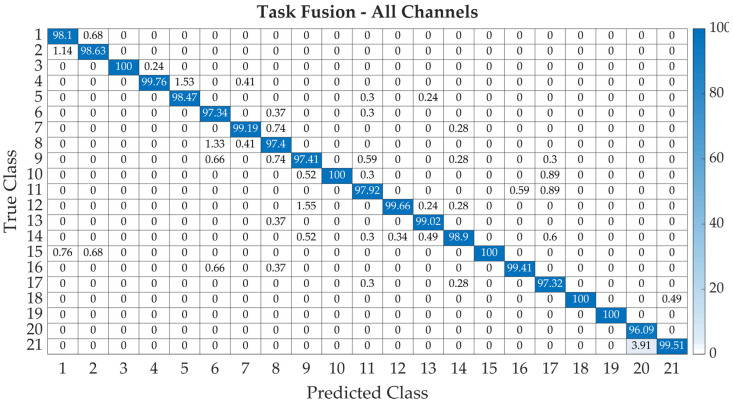
The confusion matrix of the final model’s classification accuracy for different cases considering all channels and task fusion.

As a representative example, the normalized confusion matrix for the prediction performance of eight channels, as well as task fusion, is depicted in Figure 9. The normalized confusion matrix displays the number of correctly and incorrectly classified observations for each predicted class as percentages of the number of observations of the corresponding predicted class. The diagonal cells correspond to the class-wise precision or positive predictive values. Confusion matrices for all investigated cases are enclosed in the Supplementary Material.

The overall results obtained in this study reveal that EEG signals represent an effective biometric identifier in user identification. As shown in Figure 5, Figure 6, Figure 7 and Figure 8, the mental task in the form of playing a serious game had a slightly better accuracy performance compared to the resting state paradigm. Although performance degradation was observed when combining individual game levels, task fusions, or reduction of the number of channels, the overall accuracy was still sustained at over 93%. 

A brief comparison of the proposed method within existing research works is listed in Table 8. It summarizes an overview of important information from several previous EEG-based person identification studies that utilize deep learning algorithms. Our results are challenging to compare quantitatively with others, as the EEG data used do not originate from the database investigated so far. It should be noted that we present a paradigm of playing a serious game for person identification, which has not yet been studied to the best of our knowledge. The performance of the proposed paradigm indicates competition with other state-of-the-art studies, and high person identification accuracy rate values indicate its applicability in biometric systems. Using the relatively non-complex 1D-CNN architecture, results above 99% accuracy for individual tasks and above 98% accuracy for the fusion of these tasks are achieved using only eight channels compared to other studies.

Due to the significant reduction in the number of channels (eight or four channels) and relatively high accuracy (above 98%), the EEG signal modality is a promising alternative to other biometric signals from the human body. A major advantage of using the bioelectric signal modality, such as an EEG, represents the signal’s unique patterns, which could be challenging to copy or imitate in a real-life scenario while operating an EEG-based biometrics system. The cost of this approach represents the acquisition system, namely its price and time-duration of the measurement preset and acquisition protocol. However, by using a non-research-grade EEG device, properly selected channels, and a properly designed game, the presented approach could be a cost-effective solution in terms of practical applications.

In the further continuation of this research, we plan to create a real biometric application based on the presented procedures and dataset with the possibility of expanding the number of persons. In addition, tests for the repetitive game session would be conducted to assess the stability of the EEG signals in terms of learning factors and thus demonstrate the suitability and sustainability of the proposed protocol in the identification field.

## 4. Conclusions

This study deals with person identification using the EEG signals of 21 subjects recorded during resting and while playing a serious game. The presented protocol has not been researched in the context of biometric identification before. The EEG records were divided into four task groups: a resting state with eyes closed and three game levels. Person identification was performed for each task independently, and a fusion of the three game levels and a fusion of all tasks were also created, culminating in a total of five situations. The 1D-CNN deep learning algorithm performed feature extraction in the time domain and classification. Specifically, two variants of the classification model were evaluated, the first with three and the second with four convolutional blocks, using a 5-fold CV. At the same time, the influence of the systematic reduction of the number of EEG channels was investigated, where the influence of individual scalp regions on the accuracy of classification was gradually rated. The resulting model was evaluated on a test data set. The average accuracy reached a value of more than 99% and 98% for individual tasks and task fusion, respectively. As the number of channels decreased to half, the classification accuracy also decreased to >97% for resting state, level 1, and level 2. For level 3, accuracy was ~95% and task fusion >93%. The decrease in accuracy in the case of level 3 is probably related to its incompleteness for some subjects. The use of only one scalp region was rejected because satisfactory results were not achieved.

The presented approach can provide person identification for multi-level system security and continuous person identification when a variable brain load level. A biometric system based on changing brain loads is advantageous in real-life scenarios, as a person performing various complex tasks can still be identifiable and so will not be denied access to a particular device. One of the possible approaches to the future is further implementation, such as identifying and recognizing students during online exams with the elimination of cheating or staff in the home office with different competencies.

## Figures and Tables

**Table 1:** Classification metrics for the variants of the 1D-CNN model of person identification based on resting state using 5-fold CV method (result format: mean ± standard deviation).

Channels	Variant A				Variant B			
	Average Accuracy (%)	Macro Average Precision (%)	Macro Average Recall (%)	Macro Average F1 Score (%)	Average Accuracy (%)	Macro Average Precision (%)	Macro Average Recall (%)	Macro Average F1 Score (%)
**all**	**99.17 ± 0.41**	**99.13 ± 0.37**	**99.32 ± 0.31**	**99.22 ± 0.34**	**99.46 ± 0.44**	**99.37 ± 0.61**	**99.53 ± 0.35**	**99.45 ± 0.44**
**F, C**	91.19 ± 1.29	91.43 ± 1.27	91.40 ± 1.63	91.41 ± 1.43	96.40 ± 0.85	96.47 ± 0.76	96.46 ± 0.62	96.46 ± 0.68
**F, O**	93.53 ± 1.52	93.68 ± 1.46	93.67 ± 1.27	93.67 ± 1.36	97.32 ± 0.67	97.41 ± 0.72	97.39 ± 0.59	97.40 ± 0.65
**F, P**	91.82 ± 1.25	92.10 ± 1.67	91.84 ± 1.65	91.97 ± 1.66	96.01 ± 1.94	95.93 ± 1.97	96.20 ± 1.76	96.06 ± 1.86
**C, O**	**95.18 ± 1.25**	**95.17 ± 1.22**	**95.23 ± 1.24**	**95.20 ± 1.23**	**97.52 ± 1.01**	**97.45 ± 1.02**	**97.58 ± 1.00**	**97.51 ± 1.01**
**C, P**	93.63 ± 1.02	94.04 ± 0.92	93.79 ± 1.35	93.91 ± 1.09	97.37 ± 0.92	97.31 ± 0.88	97.54 ± 0.84	97.42 ± 0.86
**P, O**	89.15 ± 2.27	89.93 ± 2.06	89.15 ± 2.13	89.54 ± 2.09	95.38 ± 1.39	95.47 ± 1.21	95.69 ± 1.37	95.58 ± 1.29
**F**	57.86 ± 2.77	56.81 ± 2.93	57.29 ± 2.51	57.05 ± 2.70	64.28 ± 3.45	63.50 ± 3.66	64.02 ± 3.48	63.76 ± 3.57
**C**	**60.19 ± 2.89**	**61.14 ± 3.88**	**59.69 ± 2.81**	**60.41 ± 3.26**	**69.83 ± 1.05**	**70.40 ± 1.20**	**69.95 ± 0.50**	**70.17 ± 0.71**
**P**	58.83 ± 4.34	61.43 ± 4.01	59.05 ± 4.35	60.22 ± 4.17	67.25 ± 1.11	68.64 ± 2.65	67.47 ± 1.41	68.05 ± 1.84
**O**	59.17 ± 2.84	58.51 ± 2.95	58.96 ± 2.48	58.73 ± 2.69	65.45 ± 2.06	65.94 ± 1.60	65.03 ± 1.26	65.48 ± 1.41

**Table 2:** Classification metrics for the variants of the 1D-CNN model of person identification based on level 1 using 5-fold CV method validation data (result format: mean ± standard deviation).

Channels	Variant A				Variant B			
	Average Accuracy (%)	Macro Average Precision (%)	Macro Average Recall (%)	Macro Average F1 Score (%)	Average Accuracy (%)	Macro Average Precision (%)	Macro Average Recall (%)	Macro Average F1 Score (%)
**all**	**98.99 ± 0.45**	**99.02 ± 0.45**	**98.92 ± 0.33**	**98.97 ± 0.38**	**99.61 ± 0.24**	**99.53 ± 0.38**	**99.57 ± 0.21**	**99.55 ± 0.27**
**F, C**	90.55 ± 1.56	90.69 ± 1.34	89.24 ± 1.79	89.96 ± 1.53	94.40 ± 1.29	94.23 ± 1.73	93.58 ± 1.39	93.90 ± 1.54
**F, O**	92.76 ± 0.90	92.36 ± 0.89	91.72 ± 0.92	92.04 ± 0.90	95.99 ± 0.54	95.49 ± 0.81	95.18 ± 0.56	95.33 ± 0.66
**F, P**	92.64 ± 0.79	92.61 ± 1.03	92.73 ± 0.86	92.67 ± 0.94	96.11 ± 0.68	95.88 ± 0.91	95.98 ± 0.86	95.93 ± 0.88
**C, O**	**94.21 ± 1.32**	**93.87 ± 1.50**	**93.62 ± 1.66**	**93.74 ± 1.58**	96.50 ± 0.86	96.49 ± 0.94	96.07 ± 1.12	96.28 ± 1.02
**C, P**	92.93 ± 0.92	93.53 ± 0.85	93.14 ± 0.91	93.33 ± 0.88	**96.71 ± 0.67**	**96.81 ± 0.95**	**96.75 ± 0.40**	**96.78 ± 0.56**
**P, O**	91.60 ± 0.97	91.84 ± 1.09	90.19 ± 1.05	91.01 ± 1.07	95.45 ± 0.27	95.47 ± 0.50	94.62 ± 0.49	95.04 ± 0.49
**F**	56.05 ± 1.74	51.55 ± 2.15	49.87 ± 1.65	50.70 ± 1.87	61.22 ± 1.80	59.36 ± 1.53	56.17 ± 2.05	57.72 ± 1.75
**C**	57.56 ± 1.94	56.52 ± 1.93	53.62 ± 2.13	55.03 ± 2.03	64.10 ± 1.56	62.56 ± 0.98	59.80 ± 0.92	61.15 ± 0.95
**P**	46.82 ± 3.35	45.46 ± 3.64	40.92 ± 3.19	43.07 ± 3.40	48.05 ± 3.86	48.76 ± 2.62	42.24 ± 4.39	45.27 ± 3.28
**O**	**58.61 ± 2.83**	**57.71 ± 1.92**	**53.25 ± 2.46**	**55.39 ± 2.16**	**64.88 ± 1.50**	**64.93 ± 0.89**	**60.47 ± 1.07**	**62.62 ± 0.97**

**Table 3:** Classification metrics for the variants of the 1D-CNN model of person identification based on level 2 using 5-fold CV method validation data (result format: mean ± standard deviation).

Channels	Variant A				Variant B			
	Average Accuracy (%)	Macro Average Precision (%)	Macro Average Recall (%)	Macro Average F1 Score (%)	Average Accuracy (%)	Macro Average Precision (%)	Macro Average Recall (%)	Macro Average F1 Score (%)
**all**	**99.41 ± 0.41**	**99.42 ± 0.49**	**99.24 ± 0.57**	**99.33 ± 0.53**	**99.72 ± 0.13**	**99.67 ± 0.17**	**99.72 ± 0.18**	**99.69 ± 0.17**
**F, C**	87.73 ± 1.49	89.05 ± 0.98	85.78 ± 2.18	87.38 ± 1.35	93.53 ± 0.72	93.69 ± 0.59	92.27 ± 1.34	92.97 ± 0.82
**F, O**	93.31 ± 1.01	92.63 ± 1.24	92.50 ± 1.76	92.56 ± 1.45	97.32 ± 0.68	97.05 ± 0.81	97.07 ± 0.94	97.06 ± 0.87
**F, P**	90.39 ± 1.88	90.52 ± 1.51	90.17 ± 2.18	90.34 ± 1.78	96.21 ± 1.22	95.68 ± 1.17	96.07 ± 1.38	95.87 ± 1.27
**C, O**	**95.62 ± 0.78**	**94.98 ± 0.86**	**94.78 ± 1.05**	**94.88 ± 0.95**	**97.50 ± 0.73**	**97.42 ± 0.75**	**96.90 ± 1.22**	**97.16 ± 0.93**
**C, P**	94.02 ± 1.44	94.17 ± 1.61	93.13 ± 1.17	93.65 ± 1.36	97.44 ± 0.77	97.45 ± 0.71	97.12 ± 1.34	97.28 ± 0.93
**P, O**	88.69 ± 1.64	88.49 ± 1.34	87.45 ± 1.23	87.97 ± 1.28	94.98 ± 1.80	94.41 ± 1.85	94.35 ± 2.45	94.38 ± 2.11
**F**	39.69 ± 4.03	39.58 ± 2.05	37.39 ± 3.56	38.45 ± 2.60	45.61 ± 6.16	49.87 ± 6.48	44.87 ± 6.99	47.24 ± 6.73
**C**	57.53 ± 1.28	57.53 ± 5.04	50.15 ± 0.86	53.59 ± 1.47	65.27 ± 2.47	63.87 ± 4.07	60.81 ± 2.79	62.30 ± 3.31
**P**	25.92 ± 2.32	27.33 ± 4.52	22.51 ± 2.65	24.69 ± 3.34	26.41 ± 4.15	29.97 ± 3.44	24.23 ± 3.77	26.80 ± 3.60
**O**	**61.42 ± 2.05**	**60.23 ± 3.01**	**55.27 ± 2.02**	**57.64 ± 2.42**	**67.80 ± 1.71**	**65.04 ± 1.61**	**62.97 ± 2.51**	**63.99 ± 1.96**

**Table 4:** Classification metrics for the variants of the 1D-CNN model of person identification based on level 3 using 5-fold CV method validation data (result format: mean ± standard deviation).

Channels	Variant A				Variant B			
	Average Accuracy (%)	Macro Average Precision (%)	Macro Average Recall (%)	Macro Average F1 Score (%)	Average Accuracy (%)	Macro Average Precision (%)	Macro Average Recall (%)	Macro Average F1 Score (%)
**all**	**98.33 ± 0.18**	**98.29 ± 0.19**	**98.15 ± 0.25**	**98.22 ± 0.22**	**99.20 ± 0.16**	**99.07 ± 0.24**	**99.11 ± 0.19**	**99.09 ± 0.21**
**F, C**	89.40 ± 0.10	89.14 ± 0.60	86.73 ± 0.98	87.92 ± 0.74	93.71 ± 0.57	93.19 ± 0.78	92.53 ± 0.70	92.86 ± 0.74
**F, O**	90.57 ± 1.04	90.74 ± 0.79	89.62 ± 1.05	90.18 ± 0.90	93.80 ± 0.96	93.83 ± 0.77	93.33 ± 1.16	93.58 ± 0.93
**F, P**	92.58 ± 0.88	92.27 ± 0.49	92.05 ± 0.96	92.16 ± 0.65	**95.84 ± 0.45**	**95.81 ± 0.80**	**95.31 ± 0.72**	**95.56 ± 0.76**
**C, O**	90.52 ± 0.64	91.18 ± 0.73	89.81 ± 1.10	90.49 ± 0.88	93.83 ± 0.79	94.17 ± 0.71	93.36 ± 0.81	93.76 ± 0.76
**C, P**	**93.14 ± 0.83**	**92.73 ± 1.18**	**92.87 ± 1.32**	**92.80 ± 1.25**	95.68 ± 0.52	95.65 ± 0.59	95.57 ± 0.68	95.61 ± 0.63
**P, O**	90.31 ± 0.71	91.10 ± 0.62	88.95 ± 0.70	90.01 ± 0.66	93.98 ± 1.01	94.24 ± 1.10	93.06 ± 1.33	93.65 ± 1.20
**F**	53.41 ± 1.82	49.70 ± 1.76	47.92 ± 1.93	48.79 ± 1.84	59.86 ± 0.91	57.52 ± 0.57	55.21 ± 0.77	56.34 ± 0.66
**C**	**57.38 ± 1.78**	**57.60 ± 2.34**	**52.59 ± 1.19**	**54.98 ± 1.58**	**64.80 ± 0.92**	**64.23 ± 1.47**	**60.10 ± 1.02**	**62.10 ± 1.20**
**P**	48.02 ± 2.84	51.53 ± 2.69	44.25 ± 2.70	47.61 ± 2.69	54.13 ± 3.36	55.60 ± 3.36	49.83 ± 3.17	52.56 ± 3.26
**O**	53.53 ± 1.34	56.26 ± 0.94	50.53 ± 2.29	53.24 ± 1.33	57.61 ± 0.81	59.87 ± 0.77	55.67 ± 0.74	57.69 ± 0.75

**Table 5:** Classification metrics for the variants of the 1D-CNN model of person identification based on level fusion (playing game sequence) using 5-fold CV method validation data (result format: mean ± standard deviation).

Channels	Variant A				Variant B			
	Average Accuracy (%)	Macro Average Precision (%)	Macro Average Recall (%)	Macro Average F1 Score (%)	Average Accuracy (%)	Macro Average Precision (%)	Macro Average Recall (%)	Macro Average F1 Score (%)
**all**	**97.84 ± 0.18**	**97.63 ± 0.13**	**97.66 ± 0.31**	**97.64 ± 0.18**	**98.82 ± 0.29**	**98.65 ± 0.26**	**98.77 ± 0.30**	**98.71 ± 0.28**
**F, P**	88.74 ± 0.71	88.70 ± 0.63	88.12 ± 0.64	88.41 ± 0.63	**92.58 ± 0.49**	**92.27 ± 0.57**	**92.09 ± 0.46**	**92.18 ± 0.51**
**C, O**	89.76 ± 0.87	89.70 ± 0.86	89.18 ± 1.19	89.44 ± 1.00	91.99 ± 0.67	91.81 ± 0.40	91.50 ± 0.70	91.65 ± 0.51
**C, P**	**89.88 ± 1.06**	**89.45 ± 0.91**	**89.46 ± 1.09**	**89.45 ± 0.99**	92.54 ± 0.59	92.11 ± 0.83	92.12 ± 0.86	92.11 ± 0.84

**Table 6:** Classification metrics for the variants of the 1D-CNN model of person identification based on all task fusion (resting state + playing game sequence) using 5-fold CV method validation data (result format: mean ± standard deviation).

Channels	Variant A				Variant B			
	Average Accuracy (%)	Macro Average Precision (%)	Macro Average Recall (%)	Macro Average F1 Score (%)	Average Accuracy (%)	Macro Average Precision (%)	Macro Average Recall (%)	Macro Average F1 Score (%)
**all**	**98.08 ± 0.30**	**98.06 ± 0.29**	**98.04 ± 0.32**	**98.05 ± 0.30**	**98.97 ± 0.12**	**98.91 ± 0.18**	**98.93 ± 0.10**	**98.92 ± 0.13**
**F, P**	89.11 ± 0.58	89.30 ± 0.76	88.64 ± 0.70	88.97 ± 0.73	92.40 ± 0.49	92.39 ± 0.44	91.99 ± 0.60	92.19 ± 0.51
**C, O**	89.54 ± 0.59	89.54 ± 0.62	88.88 ± 0.58	89.21 ± 0.60	92.22 ± 0.86	92.15 ± 0.94	91.94 ± 0.80	92.04 ± 0.86
**C, P**	**91.01 ± 0.77**	**90.75 ± 0.62**	**90.68 ± 0.73**	**90.71 ± 0.67**	**93.39 ± 0.42**	**93.17 ± 0.45**	**93.09 ± 0.40**	**93.13 ± 0.42**

**Table 7:** Classification metrics for the evaluation of the final model of person identification based on individual tasks and task fusion test data.

Channels	Task	Average Accuracy (%)	Macro Average Precision (%)	Macro Average Recall (%)	Macro Average F1 Score (%)
**All**	Resting State	99.80	99.78	99.82	99.80
	Level 1	99.77	99.67	99.70	99.68
	Level 2	99.88	99.83	99.74	99.78
	Level 3	99.04	99.00	99.09	99.04
	Game	98.79	98.79	98.72	98.75
	Task fusion	98.75	98.77	98.68	98.72
**Reduced**	Resting State (C, O)	97.45	97.68	97.35	97.51
	Level 1 (C, P)	97.29	97.65	97.64	97.64
	Level 2 (C, O)	97.78	97.55	97.24	97.39
	Level 3 (F, P)	95.74	95.97	95.28	95.62
	Game (F, P)	93.33	93.17	93.17	93.17
	Task Fusion (C, P)	93.84	93.68	93.39	93.53

**Table 8:** The comparison of state-of-the-art deep learning algorithms for EEG-based biometry.

Ref.	Paradigm	Database	No. of Subjects	No. of Channels	Segment Length	Classifier, Result
[37]	Resting state	Physionet	109	14 reduced	0.5 s	2D-CNN 99.32%
[38]	Resting state, opening, and closing fists and feet both physically and imaginarily	Physionet	109	16 reduced	1 s	1D-CNN LSTM 99.58%
[56]	Resting state	Physionet	109	64	12 s	1D-CNN 99.81%
[57]	Watching film clips	DREAMER	23	14	1 s	CNN 94.01%
[58]	Signed subject signatures on mobile phone screen	Own	33 genuine and 25 forged users	14	-	BLSTM-NN 98.78%
[59]	Watching affective elicited music videos	DEAP	32	5 reduced	1 s	CNN-GRU 99.17% (CRR)
[60]	Eyes close, open, motor speech imaginarily, visual stimulation, mathematical calculation	Own	45	19	5 s	1D-CNN 95.2% (eyes open)
[61]	Photic stimulation	Own	16	16	3 s	1D-CNN 97.17%
[62]	Steady-state visual-evoked potentials	Own	8	9	-	CNN 96.78%
[63]	Auditory evoked potentials	Own	20	2/1 reduced	2 s	1D-CNN LSTM 99.53% (2 channels) 96.93% (1 channel)
				8		1D-CNN
	Rest					99.80%
	L1					99.77%
	L2					99.88%
	L3					99.04%
	GAME					98.79%
	ALL					98.75%
Prop.		Own	21		1 s	
				4 reduced		1D-CNN
	Rest					97.45%
	L1					97.29%
	L2					97.78%
	L3					95.74%
	GAME					93.33%
	ALL					93.84%

## Data Availability

The data presented in this study are available on request from the corresponding author.

## Supplementary Materials

The following supporting information can be downloaded at: https://www.mdpi.com/article/10.3390/s22197154/s1, Figure S1: The confusion matrix of the final model’s classification accuracy considering all channels and resting state; Figure S2: The confusion matrix of the final model’s classification accuracy considering reduced channels and resting state; Figure S3: The confusion matrix of the final model’s classification considering all channels and Level 1; Figure S4: The confusion matrix of the final model’s classification considering reduced channels and Level 1; Figure S5: The confusion matrix of the final model’s classification considering all channels and Level 2; Figure S6: The confusion matrix of the final model’s classification considering reduced channels and Level 2; Figure S7: The confusion matrix of the final model’s classification considering all channels and Level 3; Figure S8: The confusion matrix of the final model’s classification considering reduced channels and Level 3; Figure S9: The confusion matrix of the final model’s classification considering all channels and all game levels; Figure S10: The confusion matrix of the final model’s classification considering reduced channels and all game levels; Figure S11: The confusion matrix of the final model’s classification accuracy for different cases considering all channels and task fusion; Figure S12: The confusion matrix of the final model’s classification accuracy for different cases considering reduced channels and task fusion.
