# Supplementary material for: EEG-Based Person Identification during Escalating Cognitive Load
Source: Sensors (Basel). 2022 Sep 21;22(19):7154. doi: 10.3390/s22197154 (PMC9572021; doi:10.3390/s22197154)
Supplement: Supplementary file 1 [file sensors-22-07154-s001.zip › sensors-1907902-supplementary.pdf]

Normalized confusion matrices for final investigated cases displaying the number of correctly and incorrectly classified observations for each predicted class as percentages of the number of observations of the corresponding predicted class. The diagonal cells correspond to the class-wise precision or positive predictive values.

Resting State - All Channels

True Class \ Predicted Class	1	2	3	4	5	6	7	8	9	10	11	12	13	14	15	16	17	18	19	20	21
1	100	0	4.55	0	0	0	0	0	0	0	0	0	0	0	0	0	0	0	0	0	0
2	0	100	0	0	0	0	0	0	0	0	0	0	0	0	0	0	0	0	0	0	0
3	0	0	95.45	0	0	0	0	0	0	0	0	0	0	0	0	0	0	0	0	0	0
4	0	0	0	100	0	0	0	0	0	0	0	0	0	0	0	0	0	0	0	0	0
5	0	0	0	0	100	0	0	0	0	0	0	0	0	0	0	0	0	0	0	0	0
6	0	0	0	0	0	100	0	0	0	0	0	0	0	0	0	0	0	0	0	0	0
7	0	0	0	0	0	0	100	0	0	0	0	0	0	0	0	0	0	0	0	0	0
8	0	0	0	0	0	0	0	100	0	0	0	0	0	0	0	0	0	0	0	0	0
9	0	0	0	0	0	0	0	0	100	0	0	0	0	0	0	0	0	0	0	0	0
10	0	0	0	0	0	0	0	0	0	100	0	0	0	0	0	0	0	0	0	0	0
11	0	0	0	0	0	0	0	0	0	0	100	0	0	0	0	0	0	0	0	0	0
12	0	0	0	0	0	0	0	0	0	0	0	100	0	0	0	0	0	0	0	0	0
13	0	0	0	0	0	0	0	0	0	0	0	0	100	0	0	0	0	0	0	0	0
14	0	0	0	0	0	0	0	0	0	0	0	0	0	100	0	0	0	0	0	0	0
15	0	0	0	0	0	0	0	0	0	0	0	0	0	0	100	0	0	0	0	0	0
16	0	0	0	0	0	0	0	0	0	0	0	0	0	0	0	100	0	0	0	0	0
17	0	0	0	0	0	0	0	0	0	0	0	0	0	0	0	0	100	0	0	0	0
18	0	0	0	0	0	0	0	0	0	0	0	0	0	0	0	0	0	100	0	0	0
19	0	0	0	0	0	0	0	0	0	0	0	0	0	0	0	0	0	0	100	0	0
20	0	0	0	0	0	0	0	0	0	0	0	0	0	0	0	0	0	0	0	100	0
21	0	0	0	0	0	0	0	0	0	0	0	0	0	0	0	0	0	0	0	0	100

Figure S1. The confusion matrix of the final model's classification accuracy considering all channels and resting state.

Resting State - Reduced Channels

True Class \ Predicted Class	1	2	3	4	5	6	7	8	9	10	11	12	13	14	15	16	17	18	19	20	21
1	95.24	0	0	0	0	5.26	0	15.38	0	0	0	0	0	0	0	0	0	0	0	0	0
2	0	100	0	0	0	0	0	0	0	0	0	0	0	0	0	0	0	0	0	0	0
3	0	0	100	0	0	0	0	0	0	0	0	0	0	0	0	0	0	0	0	0	0
4	4.76	0	0	96.3	0	0	0	0	0	0	0	0	0	0	0	0	0	0	0	0	0
5	0	0	0	0	100	0	0	0	0	0	0	0	0	0	0	0	0	0	0	0	0
6	0	0	0	0	0	94.74	0	0	0	0	0	0	0	0	0	0	0	0	0	0	0
7	0	0	0	0	0	0	100	0	0	0	0	0	0	0	0	0	0	0	0	0	0
8	0	0	0	0	0	0	0	80.77	0	0	0	0	0	0	0	0	0	0	0	0	0
9	0	0	0	0	0	0	0	0	93.75	0	0	0	0	0	0	0	0	0	0	0	0
10	0	0	0	0	0	0	0	0	0	100	0	0	0	0	0	0	0	0	0	0	0
11	0	0	0	0	0	0	0	0	0	0	100	0	0	0	0	0	0	0	0	0	0
12	0	0	0	0	0	0	0	0	0	0	0	100	0	0	0	0	3.13	0	0	0	0
13	0	0	0	0	0	0	0	0	0	0	0	0	100	0	0	0	0	0	0	0	0
14	0	0	0	0	0	0	0	3.85	6.25	0	0	0	0	100	0	0	3.13	0	0	0	0
15	0	0	0	3.7	0	0	0	0	0	0	0	0	0	0	100	0	0	0	0	0	0
16	0	0	0	0	0	0	0	0	0	0	0	0	0	0	0	100	3.13	0	0	0	0
17	0	0	0	0	0	0	0	0	0	0	0	0	0	0	0	0	90.63	0	0	0	0
18	0	0	0	0	0	0	0	0	0	0	0	0	0	0	0	0	0	100	0	0	0
19	0	0	0	0	0	0	0	0	0	0	0	0	0	0	0	0	0	0	100	0	0
20	0	0	0	0	0	0	0	0	0	0	0	0	0	0	0	0	0	0	0	100	0
21	0	0	0	0	0	0	0	0	0	0	0	0	0	0	0	0	0	0	0	0	100

Figure S2. The confusion matrix of the final model's classification accuracy considering reduced channels and resting state.

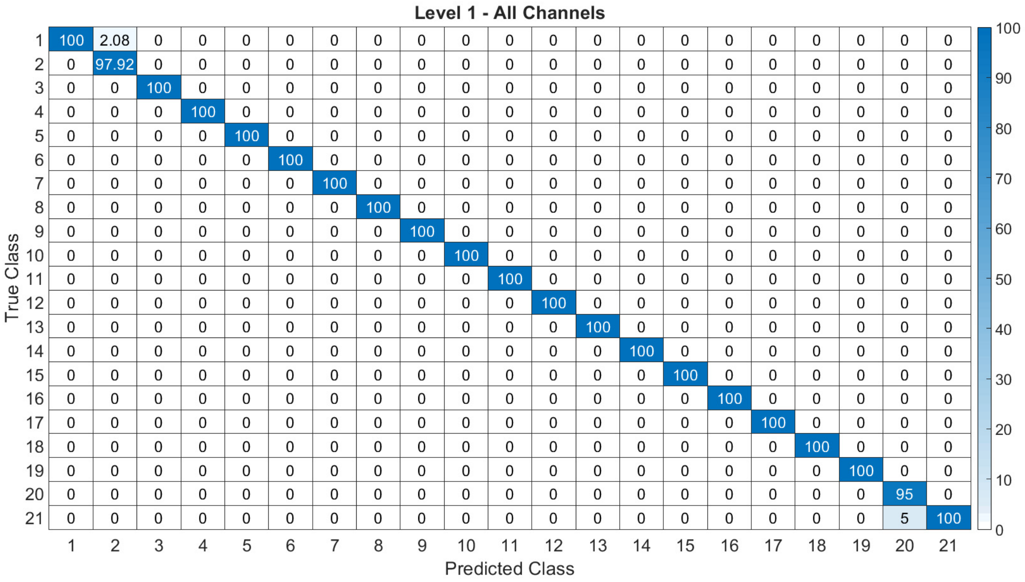

Figure S3. The confusion matrix of the final model's classification considering all channels and Level 1.

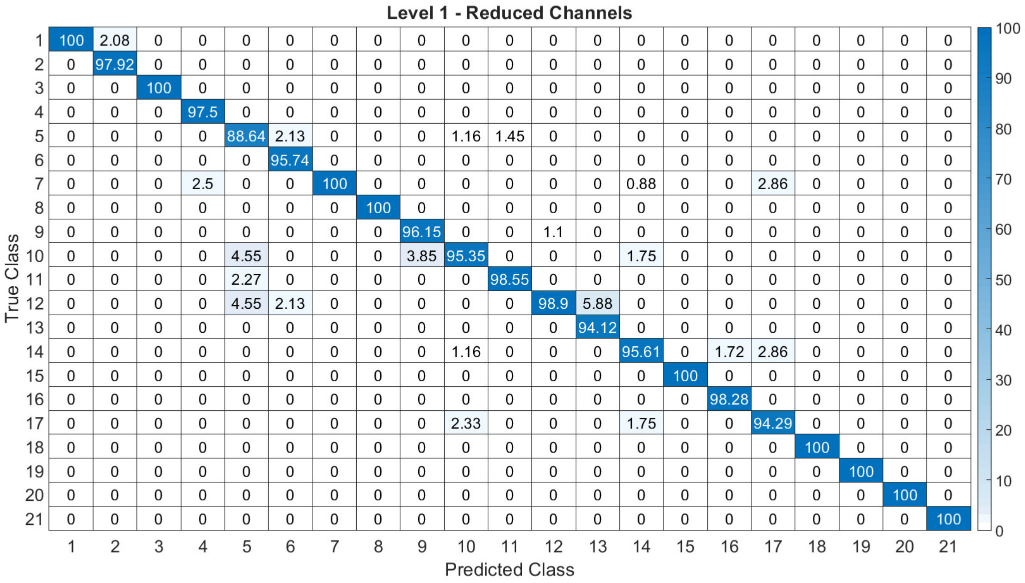

Figure S4. The confusion matrix of the final model's classification considering reduced channels and Level 1.

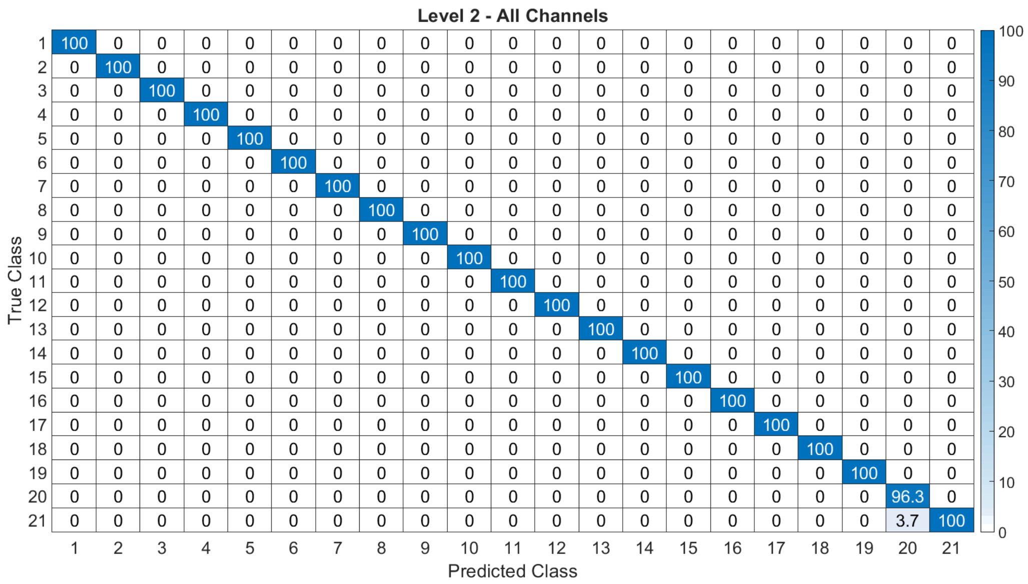

Figure S5. The confusion matrix of the final model's classification considering all channels and Level 2.

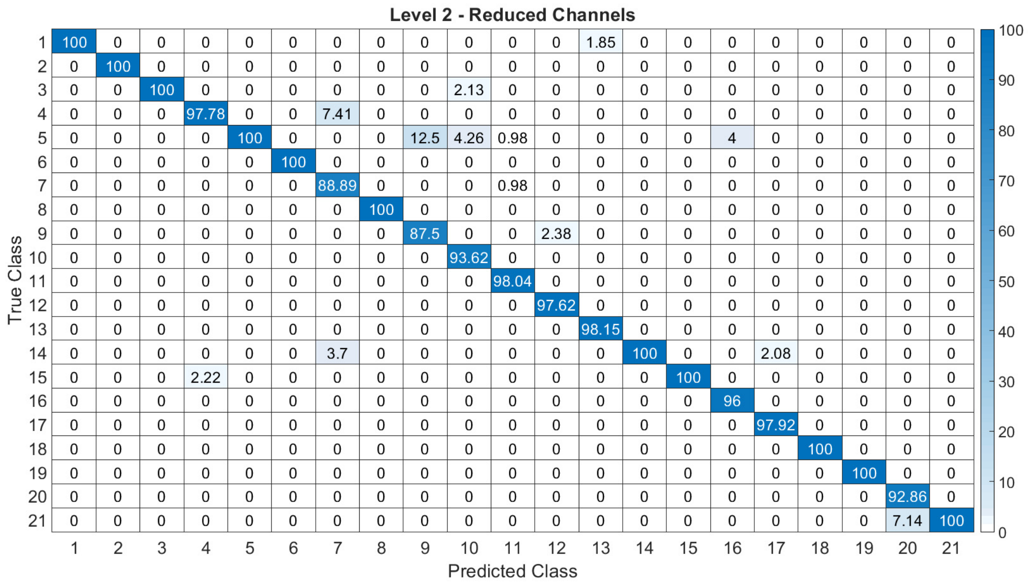

Figure S6. The confusion matrix of the final model's classification considering reduced channels and Level 2.

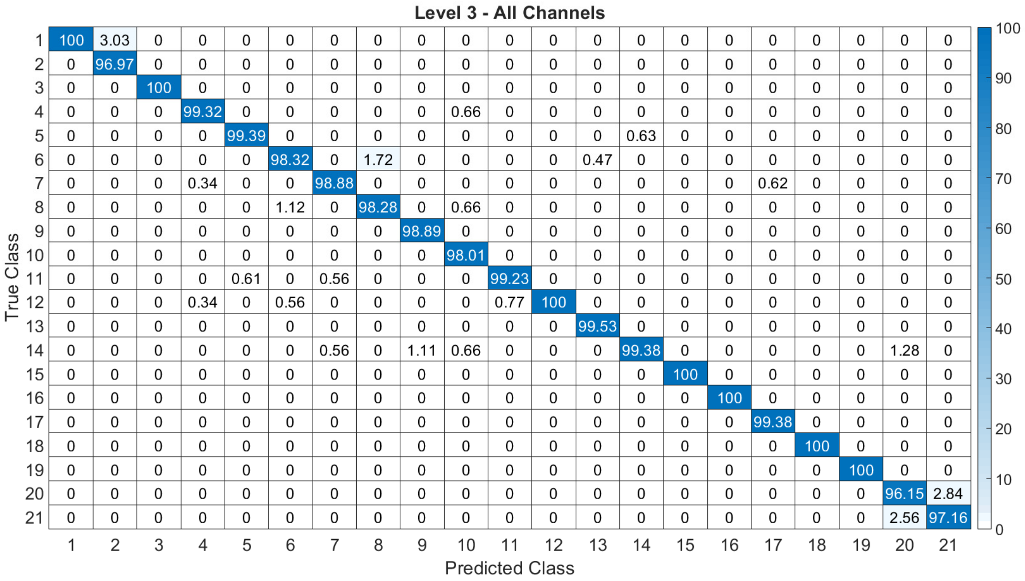

Figure S7. The confusion matrix of the final model's classification considering all channels and Level 3.

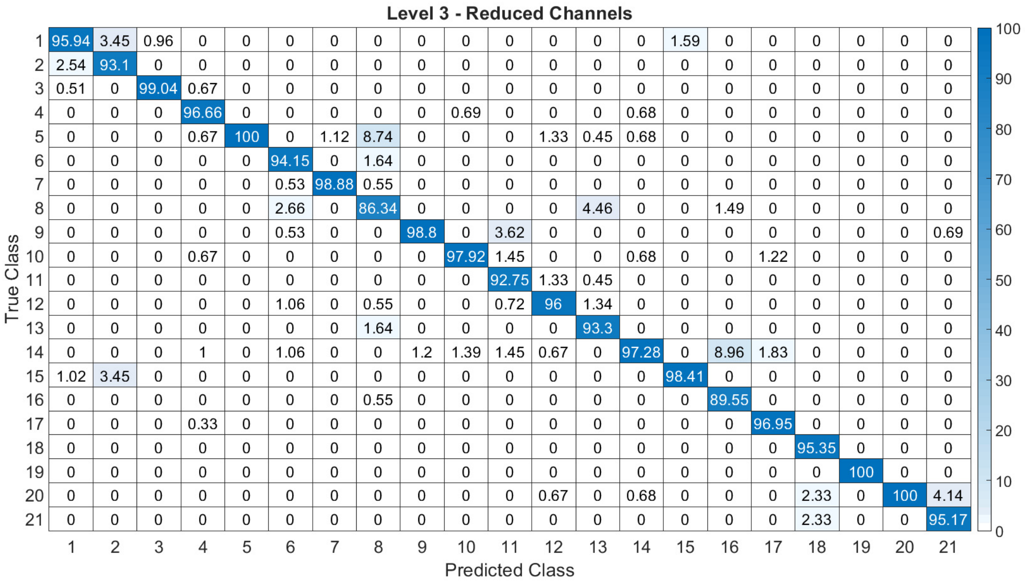

Figure S8. The confusion matrix of the final model's classification considering reduced channels and Level 3.

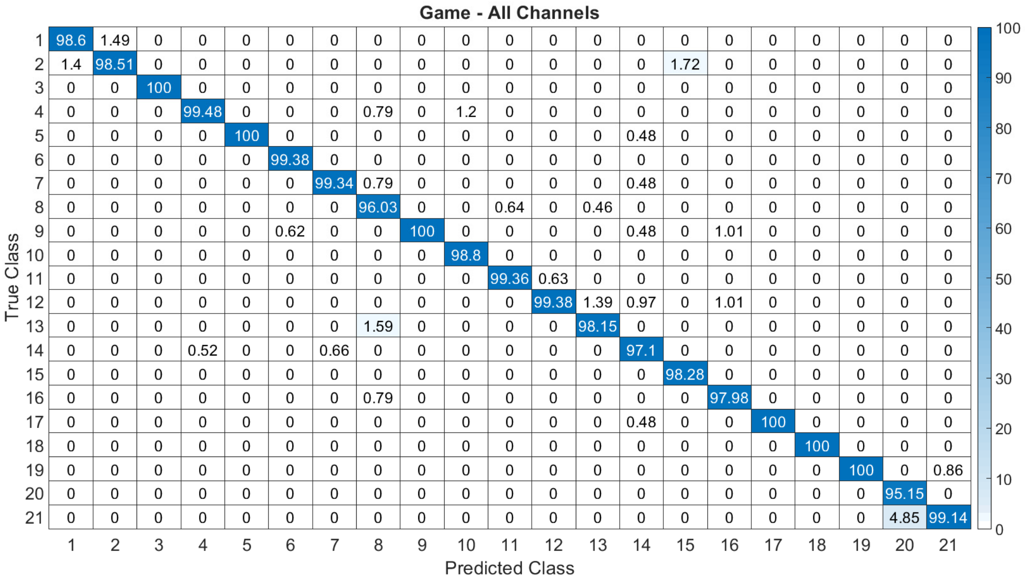

Figure S9. The confusion matrix of the final model's classification considering all channels and all game levels.

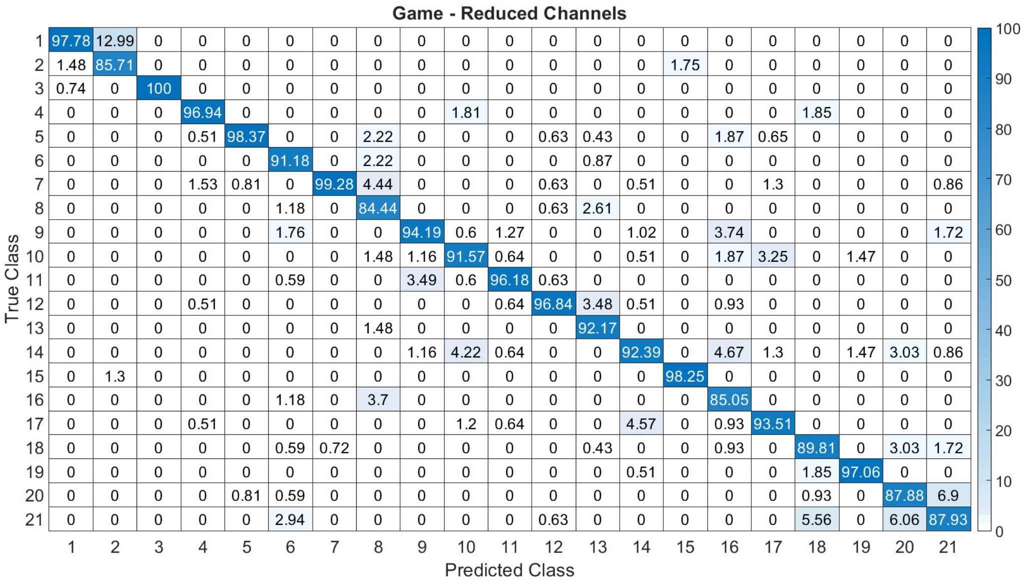

Figure S10. The confusion matrix of the final model's classification considering reduced channels and all game levels.

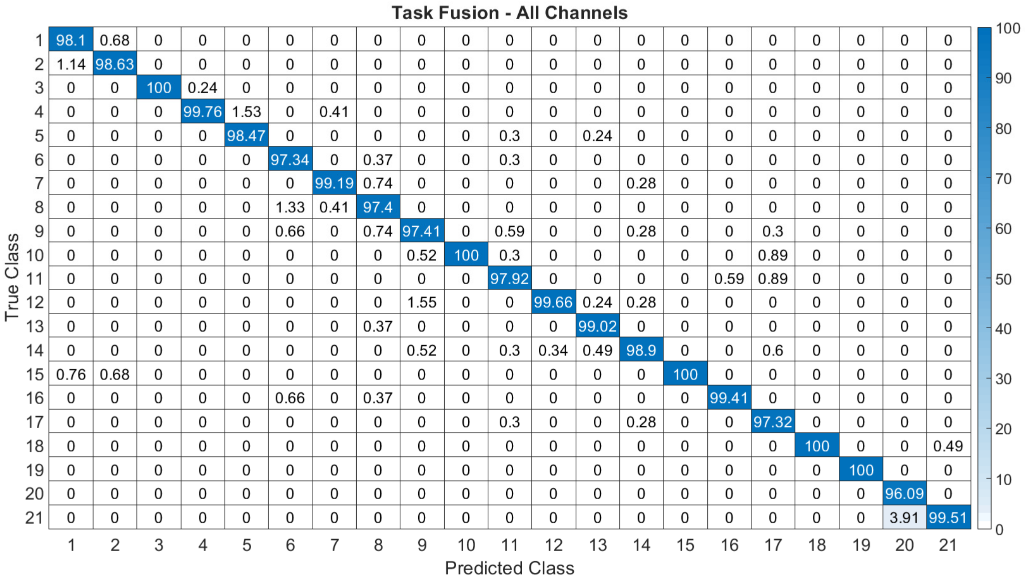

Figure S11. The confusion matrix of the final model's classification accuracy for different cases considering all channels and task fusion.

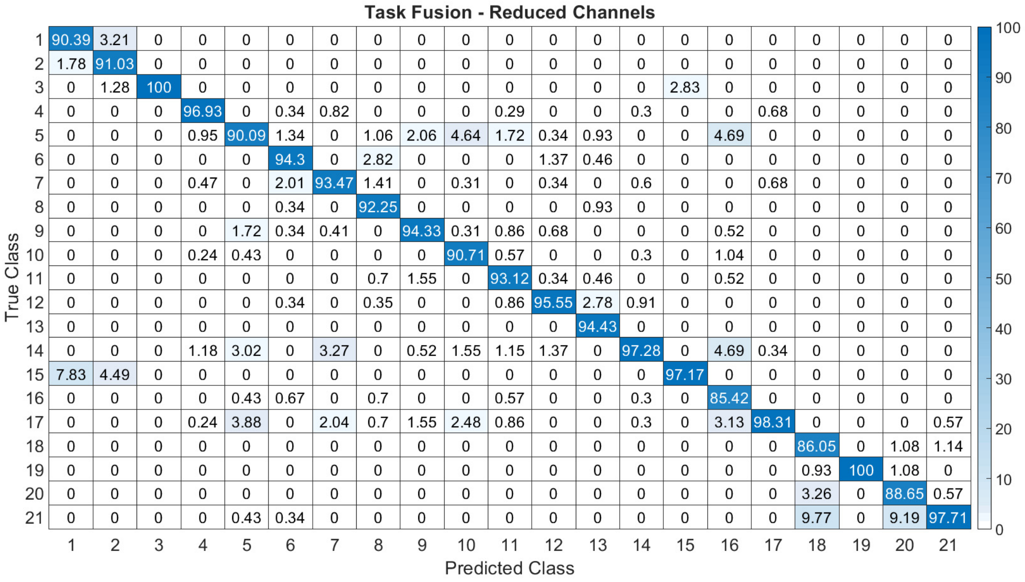

Figure S12. The confusion matrix of the final model's classification accuracy for different cases considering reduced channels and task fusion.
